# DNA Methylation Alterations at 5′-CCGG Sites in the Interspecific and Intraspecific Hybridizations Derived from *Brassica rapa* and *B. napus*

**DOI:** 10.1371/journal.pone.0065946

**Published:** 2013-06-18

**Authors:** Wanshan Xiong, Xiaorong Li, Donghui Fu, Jiaqin Mei, Qinfei Li, Guanyuan Lu, Lunwen Qian, Yin Fu, Joseph Onwusemu Disi, Jiana Li, Wei Qian

**Affiliations:** 1 College of Agronomy and Biotechnology, Southwest University, Chongqing, China; 2 College of Pharmaceutical Science, Southwest University, Chongqing, China; 3 Oil Crop Institute, China Academy of Agricultural Science, Wuhan, China; National Taiwan University, Taiwan

## Abstract

DNA methylation is an important regulatory mechanism for gene expression that involved in the biological processes of development and differentiation in plants. To investigate the association of DNA methylation with heterosis in *Brassica*, a set of intraspecific hybrids in *Brassica rapa* and *B. napus* and interspecific hybrids between *B. rapa* and *B. napus*, together with parental lines, were used to monitor alterations in cytosine methylation at 5′-CCGG sites in seedlings and buds by methylation-sensitive amplification polymorphism analysis. The methylation status of approximately a quarter of the methylation sites changed between seedlings and buds. These alterations were related closely to the genomic structure and heterozygous status among accessions. The methylation status in the majority of DNA methylation sites detected in hybrids was the same as that in at least one of the parental lines in both seedlings and buds. However, the association between patterns of cytosine methylation and heterosis varied among different traits and between tissues in hybrids of *Brassica*, although a few methylation loci were associated with heterosis. Our data suggest that changes in DNA methylation at 5′-CCGG sites are not associated simply with heterosis in the interspecific and intraspecific hybridizations derived from *B. rapa* and *B. napus*.

## Introduction

Although heterosis has been utilized widely to improve seed yield potential in crops, the underlying biological mechanisms remain poorly understood. Some efforts have been made to investigate the relationship between heterosis and parental diversity [1], and to dissect heterosis by mapping quantitative trait loci in segregating populations [2]–[5].

The differences that exist between hybrids and their parents at the level of gene expression have received considerable attention in recent years. It has been proposed that nonadditive patterns of gene expression are predominant in hybrids [6]–[8]. However, reports showed that the majority of genes that are expressed differentially in inbred parents have an additive pattern of expression in hybrids, and that novel patterns of gene expression in hybrids are rare [9], and that the proportion of additively expressed genes is correlated positively with heterosis and yield in maize [10]. Moreover, Swanson-Wagner et al. [11] observed all possible modes of gene action in a global comparison of gene expression in a maize F1 hybrid and its inbred parents [11]. The varied results of those studies led Birchler et al. [12] to propose that heterosis is a complex phenotype, and that the pattern of gene expression in hybrids is not simply an additive effect of the parental alleles.

DNA methylation, which involves the conversion of cytosine to 5-methylcytosine, is an epigenetic change that does not affect DNA sequence but does influence gene expression, especially when it occurs in the promoter region of a gene [13]. DNA methylation is involved in the biological processes of development and differentiation in plants, such as plant embryogenesis, seed viability, floral development [14]–[16], as well as stress response [17].

Given the widespread and important effects of DNA methylation on gene expression, some researchers have tried to relate heterosis to the alterations of DNA methylation in hybrids relative to their parental lines [18]–[19]. Alterations in the pattern of cytosine methylation relative to the parents are evident in interspecific hybrids, including newly formed allopolyploid and introgression lines [20]–[24], and in intraspecific hybrids [25]–[26]. However, there is a fundamental problem in these studies in that it is difficult to validate the association of the alterations of DNA methylation with the observed heterosis in statistics. Comparative studies across a variety of hybrids exhibiting various degrees of heterosis were required rather than comparison of inbred plants with their corresponding hybrids [27].

*Brassica napus* (AACC, rapeseed) and *B. rapa* (AA) are two important oilseed crops in which heterosis has been utilized widely. It is easy to develop interspecific hybrids between *B. napus* and *B. rapa* without the requirement of tissue culture [28], and strong heterosis for biomass production [28], and seed yield [29]–[30] has been documented among combinations derived from *B. napus* and *B. rapa*.

To associate the changes in DNA methylation with heterosis, a set of interspecific and intraspecific hybrids and four parental accessions each of *B. napus* and *B. rapa* was evaluated for agronomic traits in two years, and the alterations of DNA methylation at 5′-CCGG sites in seedlings and buds among hybrids and parents were monitored using methylation-sensitive amplification polymorphism analysis in this study. Our data suggest that the status in most methylation sites in hybrids was the same as that in at least one of the parental lines in both seedlings and buds, and that the alterations of DNA methylation during development were associated with the genomic structure and heterozygous status among parents and hybrids. However, no direct correlation between heterosis and the alteration of DNA methylation at 5′-CCGG sites could be found in the both interspecific and intraspecific hybrids derived from *B. rapa* and *B. napus*.

## Materials and Methods

### Parents and Combinations

We used eight elite inbred lines of *Brassica* that are widely sown in China: four accessions each of *B. napus* (Xiangyou 15, Shuyou 1, Youyan 2, and Zhongshuan 9) and *B. rapa* (Hangzou Changcai, Changge Youcai, Qixingjian, and Daye Youcaibai). A complete diallel cross was performed to develop 56 combinations: 24 intraspecific hybridizations in *B. napus* and *B. rapa*, and 32 interspecific hybridizations between *B. napus* and *B. rapa*. The parental lines were considered to be pure lines because they had been selfed successively for at least seven generations before crossing.

The parents and hybrids were sown in the experimental station of Southwest University, Chongqing, China, with a randomized complete block design, 3 m^2^ per plot, in two growth seasons, 2007 and 2008. Plant density was in accordance with farming practice in the region of the Yangtze River, i.e. a row spacing of 0.3 m and 10 plants grown in short 2.5-m rows. The field was managed in the same manner as normal agricultural production fields.

### Methylation-sensitive Amplified Polymorphism Analysis

Seedlings are considered to be associated with vegetative growth, and buds with the conversion from vegetative to reproductive growth [31]. Therefore, 1-month-old seedlings and buds just before flowering were collected to monitor alterations in cytosine methylation in the global genome in *Brassica*, using methylation-sensitive amplified polymorphisms (MSAPs). These tissues were harvested and pooled from at least 10 individuals for each accession. Genomic DNA was isolated from fresh tissue with cetyltrimethyl ammonium bromide. The MSAP method was developed from technology based on amplification fragment length polymorphisms and uses a pair of isoschizomers, *Msp*I/*Hpa*II, that display differential sensitivity to methylation at the 5′-CCGG sequence sites, full methylation of the internal cytosine (cleavage by *Msp*I but not *Hpa*II), or hemimethylation of the external cytosine (cleavage by *Hpa*II but not *Msp*I) [26]. Ten combinations of MSAP primers of *EcoR* I and *Msp*I/*Hpa*II were randomly selected to detect changes in cytosine methylation at 5′-CCGG. The products of selective amplification were visualized on 6.5% polyacrylamide sequencing gels on a Licor-4300 DNA analyzer.

To explore the genetic mechanism for the alteration of cytosine methylation at 5′-CCGG during development in *Brassica*, a matrix that described the differential methylation of cytosine among accessions (with ‘1’ for alteration and ‘0’ for no change in cytosine methylation pattern from seedling to bud) was used for principal component analysis (PCA) and analysis of molecular variance (AMOVA). PCA was performed using the software NTSYS-PC [32], to evaluate genetic diversity with respect to the alterations in cytosine methylation among accessions of *Brassica*, with the genetic distance calculated on the basis of the Dice genetic similarity coefficient [33]. AMOVA was performed using the ARLEQUIN software [34], to test the genetic structure for the alterations in cytosine methylation within or between groups. The groups were sorted according to genomic structure, for example *B. rapa*, *B. napus* and interspecific hybrids, and according to heterozygous status, for example inbred line and hybrid.

### Association of Heterosis with Alterations in DNA Methylation

The DNA methylation status of the hybrids was compared with that of their parental lines with regard to the sites that were altered. Five patterns of DNA methylation status were identified. These included P1 = P2 = F1, which signified that the same alteration in DNA methylation occurred in a hybrid and both its parents, P1 = F1≠P2, P2 = F1≠P1, and P1 = P2≠F1 (i.e. the hybrid showed hypomethylation or hypermethylation). To investigate the relationship between methylation patterns in the hybrids and mid-parental heterosis (MPH), the methylation sites that were associated with heterosis were selected by using single-marker analysis in seedlings and buds. Stepwise regression analysis of the methylation patterns of these loci selected against MPH was performed at the default 0.150 level with phenotypic trait as a dependent variable and five methylation patterns as independent variables. MPH was calculated on the basis of the performance of the parents and their hybrids. Analysis of variance (ANOVA) and Pearson correlation analysis were performed using the SAS software for the traits of interest [35].

## Results

### Cytosine Methylation Status in *Brassica*

**Figure 1:**
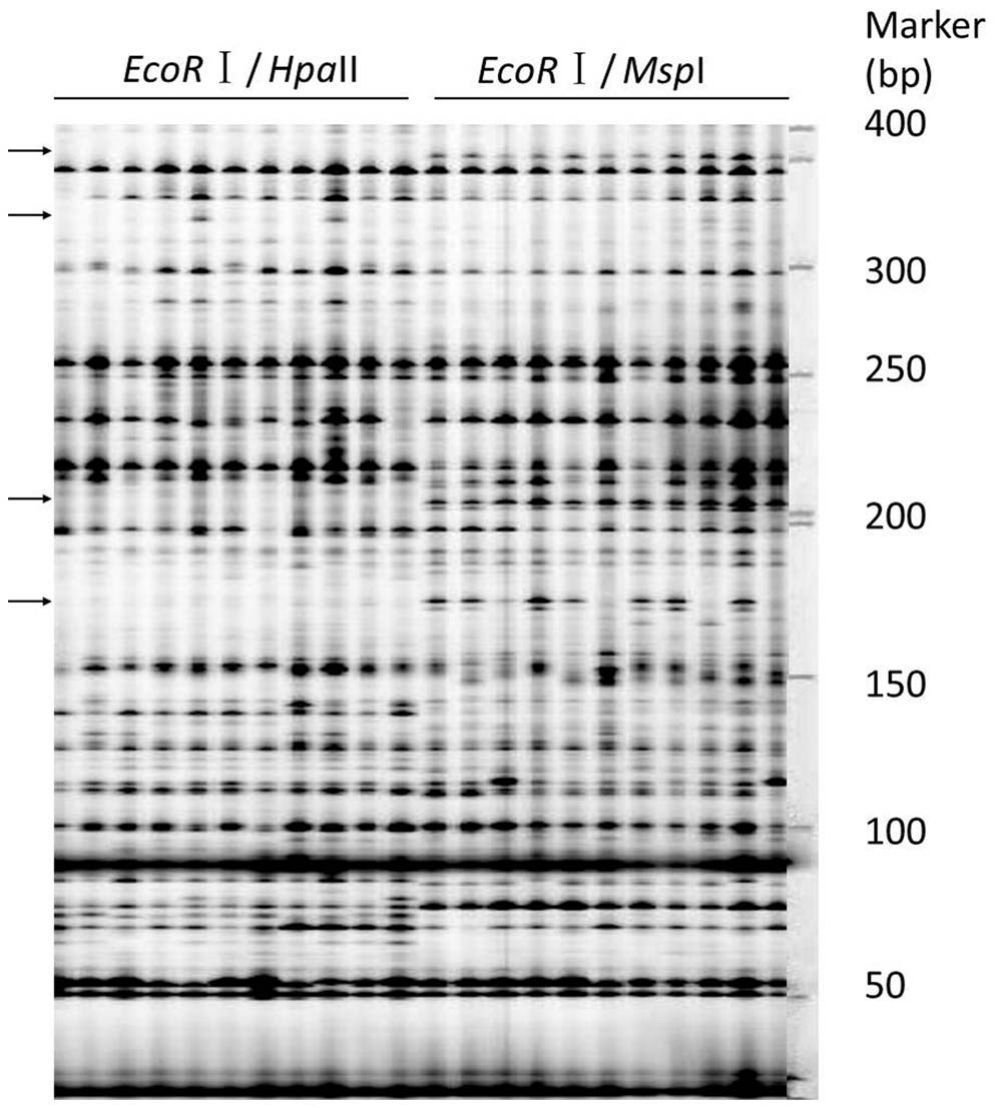
Changes in methylation-sensitive polymorphisms in bud using *EcoR* I + AAC/*Hpa*II vs *Msp*I+TAG. Arrows indicate polymorphic methylation-sensitive fragments.

We used 22 interspecific and 23 intraspecific hybrids for the MSAP analysis, together with eight parental lines. The number of available hybrids was lower than the potential total due to failure to cross or limitation of the number of seeds for some crosses. We used 10 combinations of MSAP primers to detect changes in cytosine methylation at 5′-CCGG in seedlings and buds (Fig. 1). In total, 252 polymorphism fragments were clearly detected among the 53 accessions. Of the 252 polymorphism fragments, 46 (18.2%) were amplified differentially on average in each tissue for each accession after the DNA was digested with *Msp*I and *Hpa*II.

### Tissue Specificity of Cytosine Methylation

Among the accessions in *Brassica*, seedlings and buds showed differential methylation patterns at 5′-CCGG sites, as detected by MSAP [Fig. S1]. In general, the number of sites that were amplified differentially was higher in seedlings than in buds. For seedlings, significant differences in the proportion of differentially methylated sites were detected among the 53 accessions of *Brassica*. The *B. rapa* parental lines exhibited the highest proportion of differentially methylated sites, with an average of 31.9%, followed by the *B. napus* parental lines with an average of 22.7%. The intraspecific combinations for *B. rapa* had an average value of 22.4%, whereas interspecific combinations between *B. napus* and *B. rapa* had an average value of 20.5%. The lowest proportion of differentially methylated sites was found in the intraspecific combinations for *B. napus*, with an average value of 19.9%. However, no difference was observed in the proportion of differentially methylated sites in seedlings between the interspecific and intraspecific combinations. For buds, the average proportion of differentially methylated sites was 14.5%, with no significant differences among the 53 accessions (*F* test, *P* = 0.49). And some sites that displayed the same methylation status were detected among the accessions. For example, 15 sites in seedlings and 11 in buds were detected in all eight parental lines.

Seedlings and buds are two important tissues in plant development, and are associated closely with vegetative growth and the conversion from vegetative to reproductive growth, respectively [31]. We found that the methylation status differed at approximately a quarter of the methylation sites between seedlings and buds among the 53 accessions of *Brassica*, except for the parental lines of *B. rapa* in which the status at almost half of the sites changed, and that the predominant pattern of alterations in cytosine methylation from seedlings to buds was hypomethylation over hypermethylation, except for intraspecific hybrids of *B. napus* (Table S1).

**Figure 2:**
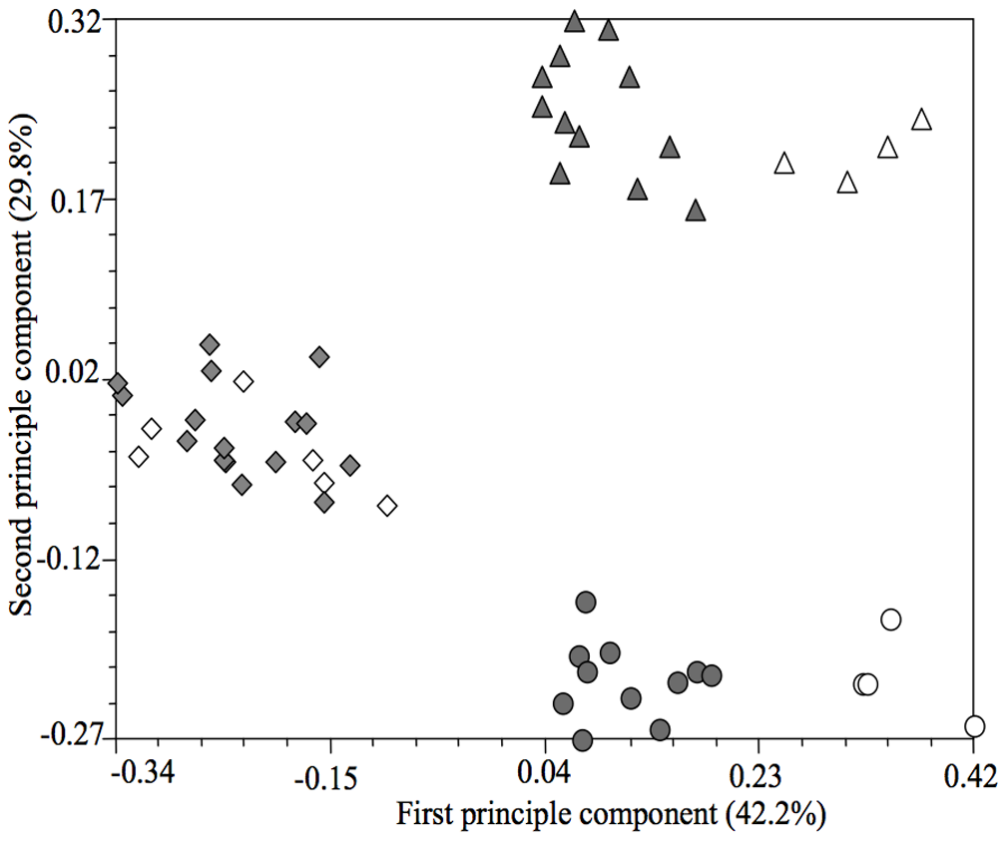
Association among eight parental lines and 45 hybrids of *Brassica* with respect to alterations in differentially methylated sites between seedlings and buds as revealed by principal component analysis. Intraspecific hybrids, *B. rapa* × *B. rapa* and *B. napus* × *B. napus*, are represented by closed triangles and closed circles in light grey, respectively. Interspecific hybrids, *B. napus* ×*B. rapa* and *B. rapa* × *B. napus* are represented by rhombuses in light grey and open rhombuses, respectively. Parental lines of *B. napus* and *B. rapa* are represented by open triangles and circles, respectively.

To explore the genetic alterations of cytosine methylation at 5′-CCGG sites during development, a matrix that described the differential status of cytosine methylation between seedlings and buds was used for PCA (Fig. 2). The total variation explained by the first and second principal components was 42.2% and 29.8%, respectively. The *Brassica* accessions could be clustered into three groups that were in accordance with the diversity of their genomic structure: *B. napus*, *B. rapa*, and interspecific hybrids between *B. napus* and *B. rapa*. Moreover, within *B. napus* and *B. rapa*, an obvious difference between hybrids and parental lines was found (Fig. 2). This indicated that heterozygous status (homozygotic *vs*. heterozygotic) influenced the alteration of cytosine methylation during development. No obvious differences in cytoplasmic effects were found in the interspecific hybrids between *B. napus* and *B. rapa,* because it was difficult to separate clearly the reciprocal crosses between *B. napus* and *B. rapa* in Fig. 2.

The importance of genomic structure and heterozygous status in the changes in cytosine methylation during development was supported by AMOVA (Table 1). Highly significant variances were found among and within genomic structures and heterozygous status (*P*≤0.001). Genomic structure accounted for 42.62% of the variation and heterozygous status for 37.79%, which indicated that both of these factors played important roles in the alteration of cytosine methylation from seedling to bud.

**Table 1:** Analysis of molecular variation of 53 accessions with different genomic structure and heterozygous status with respect to alterations in differentially methylated sites between seedlings and buds.

Group/source	df	Variance component	Variance accounted for (%)
Genomic structure			
Among genomic structures	2	11.81*	42.62
Within genomic structures	50	15.90*	57.38
Total	52	27.71	
Heterozygous status			
Among heterozygous status	1	10.75*	33.79
Within heterozygous status	51	21.06*	66.21
Total	52	31.80	

* : Significant at *P* = 0.001.

### Differential Cytosine Methylation among Parents and Hybrids

Cytosine methylation status at 5′-CCGG sites in seedlings and buds was compared between parents and hybrids. The parents exhibited more differentially methylated sites in seedlings than the hybrids did, whereas there were no significant differences between parental lines and hybrids in buds (SI Fig. 1). In general, the proportion of differentially methylated sites in a hybrid correlated positively to some extent with the average value of its two parents in both seedlings (*r* = 0.356, *P* = 0.008) and buds (*r* = 0.24, *P* = 0.056).

The patterns and extent of the alterations in cytosine methylation in hybrids relative to their parents are shown in Table 2. Approximately 85% and 91% of sites detected by MSAP in hybrids shared their cytosine methylation status with at least one of their parents in seedlings and buds, respectively. This indicated that hybrids were associated closely with their parents with regard to the alteration of cytosine methylation. The pattern of alterations in cytosine methylation at 5′-CCGG sites in interspecific hybrids between *B. napus* and *B. rapa*, especially in seedlings, showed more similarities to the pattern for parental *B. napus* than that for parental *B. rapa*, regardless of whether the former acted as the female or male parent (Table 2). This indicated that there were no obvious cytoplasm effects with respect to the extent of alteration of cytosine methylation at 5′-CCGG sites in the interspecific hybrids.

**Table 2:** Pattern and extent of alterations at differentially methylated 5′-CCGG sites among hybrids relative to parental lines in *Brassica.*

Combinations	P1 = P2 = F1 (%)	P1 = F1≠P2 (%)	P2 = F1≠P1 (%)	Hypomethylation (%)	Hypermethylation (%)
Seedling					
*B. rapa × B. napus*	56.1	9.7	22	9.6	2.5
*B. napus × B. rapa*	55.6	23	9.1	10.6	1.6
*B. rapa × B. rapa*	60	12.2	11.8	13.3	2.7
*B. napus × B. napus*	77	6.7	6.2	8.4	1.6
Bud					
*B. rapa × B. napus*	80.1	7.2	7.3	4.1	1.4
*B. napus × B. rapa*	78	9.9	6.7	4	1.4
*B. rapa × B. rapa*	78.6	7	7.1	3.9	3.4
*B. napus × B. napus*	81.8	5.1	5.3	3.1	4.6

Considering the patterns for the differentially methylated sites in hybrids relative to their parental lines, hypomethylation predominated over hypermethylation in both interspecific hybrids (10.3 *vs*. 1.9%) and intraspecific hybrids (10.8 *vs.* 2.1%) in seedlings, and in interspecific hybrids in buds (4.1 *vs.* 1.4%) (Table 2).

### Association of Cytosine Methylation with Heterosis

Seven agronomic traits were observed among the 53 accessions of *Brassica* over 2 years, and a positive correlation was found between most of these traits (Table S2). Strong MPH was seen among the hybrids for the traits observed, namely plant height (7.4%), main inflorescence length (7.1%), number of branches (6.8%), number of pods per plant (22.7%), number of seeds per pod (5.4%), seed yield (21.1%), and biomass (28.3%) (Table S3).

To reveal the relationship between methylation patterns and MPH, single-marker analysis at the 0.05 significance level was used to select 72 (28.6%) of the 252 methylation loci that were associated with heterosis (Table S4). The percentage of methylation loci associated with MPH for the seven traits was 14.4% in seedlings and 11.2% in buds on average. Of the 72 methylation loci associated with heterosis, 55 (76.4%) influenced more than one trait simultaneously in the same or reverse direction. For two traits that exhibited a high positive correlation, there were more loci that overlapped in the same direction and fewer that overlapped in a different direction (Table S4). Unfortunately, we could not obtain additional information about the association of biomass and seed yield with the other traits due to the limited amount of research samples, i.e. the intraspecific hybrids were used to evaluate seed yield rather than biomass, whereas the interspecific hybrids were used to evaluate biomass rather than seed yield.

The loci associated with heterosis were used for stepwise regression analysis of methylation patterns against heterosis (Table 3). All five methylation patterns were found to contribute to heterosis in seedlings and buds. Each methylation pattern significantly affected one or more traits, whereas each trait was associated with one or more methylation patterns. The methylation patterns that were associated with heterosis varied according to traits and tissues, and no unified methylation pattern could explain heterosis for all seven traits in both tissues (Table 3). This suggests that heterosis might not be associated simply with genome-wide alterations in DNA methylation.

**Table 3:** Impact of five methylation patterns on agronomic traits in seedlings and buds.

Pattern	Plant Height	Main Inflorescence Length	No. of Branch	No. of Podper Plant	No. of Seedper Pod	Seed Yield	Biomass
Seedling							
P1 = P2 = F1						0.36/0.26*	
P1 = F1≠P2	0.09/0.11*	0.13/0.17**	0.10/0.07		1.64/0.4***		0.47/0.08
P2 = F1≠P1			−2.4		−18.3333		0.53/0.66***
Hypomethylation				1.40/0.17**			
Hypermethylation				4.1/0.36***	5.79/0.05		
Bud							
P1 = P2 = F1				1.03/0.15**			−0.89/0.57***
P1 = F1≠P2							0.76/0.09
P2 = F1≠P1						0.49/0.07	
Hypomethylation						−2.03/0.33**	
Hypermethylation	−4.14286	−0.48/0.15*		−13.0909	−3.95/0.19**		

The loci in association with heterosis detected by using single marker analysis were used for stepwise regression analysis of methylation patterns against heterosis at the default 0.150 level. The numbers to the left and right of each slash represent the regression coefficient and coefficient of determination, respectively.

* ,**,***: p = 0.05, 0.01 and 0.001, respectively.

## Discussion

### Alterations of DNA Methylation

DNA methylation is an important aspect of epigenetics that regulates gene expression, and is involved in the biological processes of development and differentiation. In the present study, on average, a quarter of cytosine methylation sites throughout the global genome were observed to be methylated differentially between seedlings and buds among 53 accessions of *Brassica*, and that hypomethylation predominated over hypermethylation from seedlings to buds, except for intraspecific hybrids of *B. napus*. Similar tissue-specific cytosine methylation has been reported in other plants [36]–[38]. This implies that DNA methylation is tissues specific, and possibly is influenced by hybridization.

In the study reported herein, most cytosine methylation sites in interspecific and intraspecific hybrids of *Brassica* shared the same status as that of at least one of the parents. This observation was in accordance with Zhang et al. [39], who have shown that the profile of cytosine methylation in hybrids of sorghum deviates from those of their parents at a rate of only 1.69–3.22% of sites. These results have indicated that the methylation status at most cytosine methylation sites displays stable inheritance from inbred parents to hybrids. Moreover, we found that alterations in cytosine methylation status between seedling and bud were associated closely with genomic structure and heterozygous status in *Brassica*, which indicates that changes in cytosine methylation do not occur randomly during development. Similar observations have also been reported in humans by Bjornsson et al [40], who found that methylation changes over time, were clustered among families. It was reported that most methylation changes that occur in the first generation of resynthesized lines in *Brassica* can be fixed in later generations [20]. These findings show that methylation changes during development might be under genetic control, although how genetic mechanisms regulate the alteration of methylation remains to be elucidated. However, it is worth to mention that the interspecific hybrids between *B. napus* and *B. rapa*, especially in seedlings, showed more similarities to the pattern for parental *B. napus* than for parental *B. rapa* in this study, indicating that genomic asymmetry possible involves in alteration of methylation.

### Association of DNA Methylation with Heterosis

Extensive alterations in methylation have been documented in resynthesized lines in *B. napus* [20], [24], [41], *Arabidopsis* [22], and *Spartina* [23], as well as introgression lines [21]. It was proposed that alterations in cytosine methylation in interspecific hybrids are induced by hybridization [21]. If this is the case, a burst of methylation changes should be detected in F1 hybrids relative to parental lines. However, we did not detect such a burst in both interspecific and intraspecific F1 hybrids of *B. napus* and *B. rapa*, in comparison with parental lines. Even the rate of methylation changes in buds in reciprocal crosses between *B. napus* and *B. rapa* was lower than that in the parents. Udall et al. [42] have observed that *de novo* chromosomal rearrangements occur frequently in the vicinity of pre-existing translocations in resynthesized *B. napus*. This suggests that only a few rearrangements, which are produced during meiosis in S0 plants, give rise to an increasing number of genomic changes in the following generations [43]. A possible explanation is that pairing between nonhomologous chromosomes at first meiosis in an interspecific hybrid, causes genome restructuring in later generations, and that many of the meiosis-driven genetic changes are transmitted to the progeny [43], with accompanying epigenetic changes [44]. Therefore, alterations in DNA methylation in the progeny of interspecific hybrids are possibly triggered by nonhomologous gene–gene interactions, rather than by hybridization.

In this study, we found that interspecific and intraspecific hybrids of *Brassica* exhibited differential methylation status, including hypo- and hypermethylation, relative to their parental lines at 5′-CCGG methylation sites in seedlings and buds. Differential methylation status relative to the parents has also been observed at 5′-CCGG methylation sites in hybrids [21], [26].

There are three different types of DNA methylation in plant, CG, CHG, and CHH (where H is any nucleotide but G) [45]. It should be remembered in the mind that we only monitored one of DNA methylation type, the DNA methylation at 5′-CCGG sites. We could not detect any obvious correlation between heterosis and genome-wide DNA methylation at 5′-CCGG sites, because the association of patterns of cytosine methylation with heterosis varied among traits and between tissues in this study. However, Shen et al. [18] found that the genome-wide remodeling of CHH sites directed by RNA- directed DNA methylation (RdDM) pathway may play a role in heterosis. In order to answer whether heterosis associated with the different types of DNA methylation, more future studies are required.

## Supporting Information

Figure S1 **Alterations in cytosine methylation at 5′-CCGG sites as identified by 252 methylation-sensitive amplified polymorphisms among 53 accessions of** ***Brassica*** **in seedlings and buds.** The right and left vertical coordinates represent the proportion and number of methylation alterations, respectively.
(PDF)

Table S1 **Pattern and extent of alterations at differentially methylated 5′-CCGG sites from seedlings to buds among parental lines and hybrids of** ***Brassica.***
(DOC)

Table S2 **Correlation of hybrid performance (upper triangle) and number of overlapping loci associated with heterosis that were detected in two tissues (lower triangle) for seven traits.**
(DOC)

Table S3 **Mid-parent heterosis of agronomic traits among hybrids in** ***Brassica.***
(DOC)

Table S4 **Number of cytosine methylation loci in association with heterosis in seedlings and buds, which was selected from 252 loci of MSAP by using single marker analysis.**
(DOC)
