# Supplementary material for: DNA Methylation Alterations at 5′-CCGG Sites in the Interspecific and Intraspecific Hybridizations Derived from Brassica rapa and B. napus
Source: PLoS One. 2013 Jun 18;8(6):e65946. doi: 10.1371/journal.pone.0065946 (PMC3688851; doi:10.1371/journal.pone.0065946)

Supporting information (SI) Figure 1: Alterations in cytosine methylation at 5'-CCGG sites as identified by 252 methylation-sensitive amplified polymorphisms among 53 accessions of *Brassica* in seedlings and buds. The right and left vertical coordinates represent the proportion and number of methylation alterations, respectively

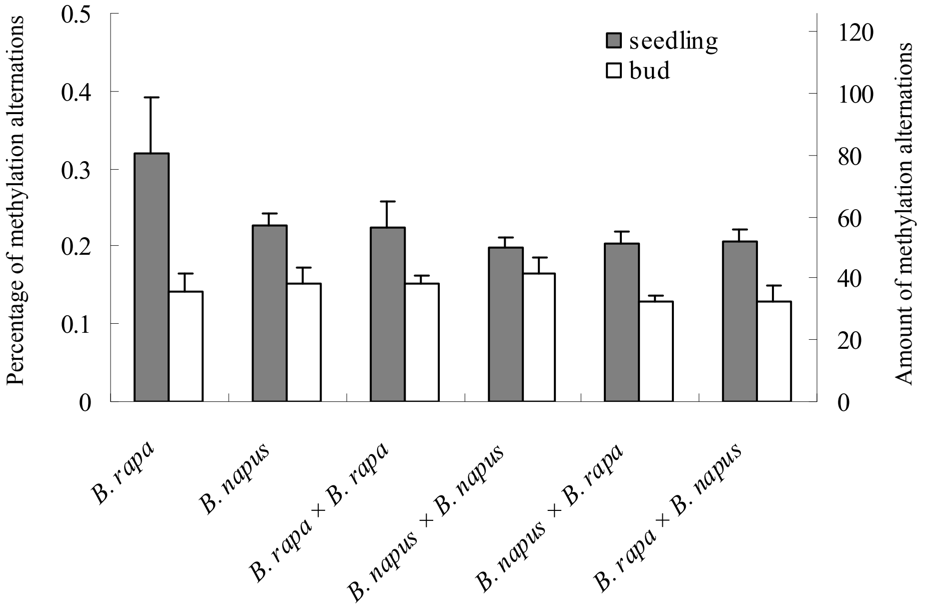

Supplement: Figure S1 — Alterations in cytosine methylation at 5′-CCGG sites as identified by 252 methylation-sensitive amplified polymorphisms among 53 accessions of Brassica in seedlings and buds. The right and left vertical coordinates represent the proportion and number of methylation alterations, respectively. (PDF) [file pone.0065946.s001.pdf]
