# Supplementary material for: DNA Methylation Alterations at 5′-CCGG Sites in the Interspecific and Intraspecific Hybridizations Derived from Brassica rapa and B. napus
Source: PLoS One. 2013 Jun 18;8(6):e65946. doi: 10.1371/journal.pone.0065946 (PMC3688851; doi:10.1371/journal.pone.0065946)
Supplement: Table S1 — Pattern and extent of alterations at differentially methylated 5′-CCGG sites from seedlings to buds among parental lines and hybrids of Brassica. (DOC) [file pone.0065946.s002.doc]

| Material | No. of accessions | Hypomethylation (%) | Hypermethylation (%) |
| --- | --- | --- | --- |
| Parents of *B. rapa* | 4 | 31.0±0.030^1^ | 14.3±0.032 |
| Parents of *B. napus* | 4 | 16.0±0.014 | 8.8±0.017 |
| *B. rapa × B. napus* | 6 | 11.0±0.017 | 9.3±0.012 |
| *B. napus × B. rapa* | 16 | 10.3±0.013 | 9.5±0.009 |
| *B. rapa × B. rapa* | 11 | 16.6±0.023 | 12.7±0.013 |
| *B. napus × B. napus* | 12 | 9.9±0.009 | 12.8±0.025 |

^1^ mean ± [standard deviation](dict://key.0895DFE8DB67F9409DB285590D870EDD/standard deviation)
