# Supplementary material for: DNA Methylation Alterations at 5′-CCGG Sites in the Interspecific and Intraspecific Hybridizations Derived from Brassica rapa and B. napus
Source: PLoS One. 2013 Jun 18;8(6):e65946. doi: 10.1371/journal.pone.0065946 (PMC3688851; doi:10.1371/journal.pone.0065946)
Supplement: Table S2 — Correlation of hybrid performance (upper triangle) and number of overlapping loci associated with heterosis that were detected in two tissues (lower triangle) for seven traits. (DOC) [file pone.0065946.s003.doc]

| Traits | 1 | 2 | 3 | 4 | 5 | 6 | 7 |
| --- | --- | --- | --- | --- | --- | --- | --- |
| Plant height (1) |  | 0.87* | 0.86* | -0.16 | 0.51* | 0.02 | 0.4 |
| Main inflorescence length (2) | 35/3^1^ |  | 0.72* | -0.11 | 0.38* | -0.07 | 0.33 |
| No. of branches (3) | 33/3 | 31/3 |  | 0.03 | 0.56* | 0.15 | 0.61* |
| No. of pods per plant (4） | 10/2 | 8/2 | 12/2 |  | -0.5* | 0.65* | 0.27 |
| No. of seeds per pod （5） | 3/0 | 4/0 | 4/1 | 0/8 |  | -0.21 | 0.21 |
| Seed yield （6） | -/- | -/- | -/- | -/- | -/- |  | -/- |
| Biomass （7） | 3/0 | 2/0 | 3/0 | 0 | 1/0 | -/- |  |

^1^: same direction / reverse direction

*: p = 0.01
