# Supplementary material for: DNA Methylation Alterations at 5′-CCGG Sites in the Interspecific and Intraspecific Hybridizations Derived from Brassica rapa and B. napus
Source: PLoS One. 2013 Jun 18;8(6):e65946. doi: 10.1371/journal.pone.0065946 (PMC3688851; doi:10.1371/journal.pone.0065946)
Supplement: Table S3 — Mid-parent heterosis of agronomic traits among hybrids in Brassica. (DOC) [file pone.0065946.s004.doc]

| Hybrids | Plant Height | Main Inflorescence Length | No. of Branch | No. of Seed per Pod | No. of Pod per Plant | Seed Yield | Biomass |
| --- | --- | --- | --- | --- | --- | --- | --- |
| *B. napus* × *B. rapa* | 11.6±6.3^﹡^ | 11.9±3.8 | 13.6±5.0 | 17.2±13.9 | -8.7±3.7 | - | 18.3±9 |
| *B. rapa* × *B. napus* | 7.0±6.4 | 6.8±5.1 | 5.7±8.1 | 31.5±30.7 | -10.2±10.7 | - | 23±16.2 |
| *B. rapa* × *B. rapa* | 2.5±7.7 | 1.7±9.4 | -0.1±8.3 | 2.3±4.9 | 2.4±6.7 | 9.5±15.6 | - |
| *B. napus* × *B. napus* | 8.1±4.1 | 7.5±4.3 | 7.8±5.9 | 6.3±4.9 | 8.5±5.6 | 15.3±22.9 | - |

^﹡^ mean ± [standard deviation](dict://key.0895DFE8DB67F9409DB285590D870EDD/standard deviation)
