# Supplementary material for: DNA Methylation Alterations at 5′-CCGG Sites in the Interspecific and Intraspecific Hybridizations Derived from Brassica rapa and B. napus
Source: PLoS One. 2013 Jun 18;8(6):e65946. doi: 10.1371/journal.pone.0065946 (PMC3688851; doi:10.1371/journal.pone.0065946)
Supplement: Table S4 — Number of cytosine methylation loci in association with heterosis in seedlings and buds, which was selected from 252 loci of MSAP by using single marker analysis. (DOC) [file pone.0065946.s005.doc]

| Trait | Seedling | Bud | Overlapping |
| --- | --- | --- | --- |
| Plant height | 22/10^1^ | 15/4^1^ | 7/3^2^ |
| Main inflorescence length | 23/10 | 15/3 | 7/3 |
| No. of branches | 21/12 | 15/3 | 7/2 |
| No. of pods per plant | 10/10 | 9/3 | 1/1 |
| No. of seeds per pod | 8/7 | 2/2 | 1/0 |
| Seed yield | 0/0 | 1/0 | 0/0 |
| Biomass | 1/0 | 1/4 | 0/0 |

^1^: positive loci/negative loci

^2^: same direction/reverse direction between seedling and bud
